# Rotavirus and *Cystoisospora suis* in piglets during the suckling and early post weaning period, in systems with solid floors and age segregated rearing

**DOI:** 10.1186/s40813-019-0114-0

**Published:** 2019-02-08

**Authors:** Emelie Pettersson, Sanna Hestad, Ivo Möttus, Eva Skiöldebrand, Per Wallgren

**Affiliations:** 1 Department of Animal Health and Antimicrobial Strategies, National Veterinary Institute, SE-751 89 Uppsala, Sweden; 2 Department of Clinical Sciences, Swedish University of Agricultural Sciences, SE-750 07 Uppsala, Sweden; 3 Bayer AB Animal Health, Gustav III: s Blvd 56, SE-169 26 Solna, Sweden; 4 Department of Biomedical Sciences and Veterinary Public Health, Swedish University of Agricultural Sciences, SE-750 07 Uppsala, Sweden

**Keywords:** Pig, Diarrhoea, Suckling, Post-weaning, Rotavirus, Cystoisospora suis, Solid floor, Age segregation

## Abstract

**Background:**

Piglet diarrhoea is considered a worldwide problem resulting in animal welfare problems and financial losses for pig farmers. Porcine rotavirus and the coccidian parasite *Cystoisospora suis* (*C. suis*) are considered two important pathogens associated with diarrhoea in piglets during the suckling and early post weaning periods. To obtain an overview on the prevalence of porcine rotavirus and *C. suis* in piglet producing herds with solid floors and age segregated rearing, faecal sampling of 791 litters in 81 farms was performed.

**Results:**

For porcine rotavirus, faecal samples were analysed using a sandwich ELISA. The overall prevalence of rotavirus in the examined herds was 11.4 ± 17.7% at 2 weeks, 56.8 ± 30.7% at 4 weeks and 71.1 ± 29.1% at 6 weeks of age and the accumulated prevalence was 49, 97 and 100%. To detect *C. suis,* faecal samples were analysed using sedimentation. The overall prevalence of *C. suis* in the examined herds was 11.9 ± 15.1% at 2 weeks of age, 10.7 ± 16.7% at 4 weeks and 8.7 ± 15.3% at 6 weeks of age and the accumulated prevalence was 56, 76 and 85%. The number of empty days between farrowing batches did influence the shedding of rotavirus at 2 weeks of age but not later. Regarding *C. suis*, no difference in prevalence was correlated to the number of days between consecutive farrowing batches.

**Conclusions:**

Our study confirmed that rotavirus should be regarded as an ubiquitous virus that can be expected to be present in almost every pig herd in Sweden. The study also demonstrated that the number of infected litters increased from birth to 6 weeks of age. Secondly, it showed that *C. suis* frequently occurred in pig herds and that the number of infected litters was rather stable from two to 6 weeks of age. Consequently, both rotavirus and *C. suis* may play a role in intestinal disturbances in piglets during the suckling and post weaning periods despite age segregated rearing, at least in systems with solid floors. However, as this study was carried out in herds without reported problems with diarrhoea or poor weight gain, the role of these pathogens should not be overestimated.

## Background

Diarrhoea in piglets during the suckling and post weaning periods is recognised as a major problem across the world, resulting in animal welfare problems and financial losses for pig farmers [1–3]. Piglet diarrhoea can have many aetiologies and after the neonatal period some of the most relevant include porcine rotavirus and the coccidian parasite *Cystoisospora suis (C. suis)* [2].

Rotavirus is an ubiquitous virus belonging to the *Reoviridae* family that may cause diarrhoea in the young of many mammals, including pigs [4]. Porcine rotavirus is prevalent in pig herds all around the world with some countries reporting 100% of the adult pigs to be seropositive [5]. Diarrhoea in piglets is most frequently caused by serogroup A rotaviruses and the highest prevalence is seen in pigs aged three to 5 weeks [3, 5]. Rotavirus serogroup B and C were previously only reported sporadically in pigs, but now serogroup C is emerging as an important cause of diarrhoea in young piglets and it is reported worldwide [4, 5]. Rotavirus replicates in the epithelial cells of mainly the jejunum and ileum, resulting in cell lysis, villous atrophy and villous blunting. This may cause malabsorption, diarrhoea and subsequent poor growth, but infections may also be subclinical [4–6].

*Cystoisospora suis* (previously known as *Isospora suis*) is a protozoan parasite belonging to the phylum apicomplexa. It is found worldwide, and infections can occur in all types of management systems [7–9]. Just like rotavirus, it is a pathogen affecting the young, and piglets become infected by ingesting sporulated oocysts from the environment. Following ingestion, sporocysts are released from the oocyst, and sporozoites are activated in the small intestine where they penetrate the epithelial cells of mainly the jejunum. The intestine becomes inflamed and macroscopically reddened. Microscopic lesions may include villous atrophy, villous fusion and crypt hyperplasia [7]. Infected animals can develop a non-haemorrhagic diarrhoea and clinical signs may occur already from 6 days of age. Most commonly, white or pasty yellow diarrhoea is seen in piglets at eight to 10 days of age [7, 10, 11]. The general condition may be affected with piglets becoming stunted. Diarrhoea may result in dehydration, but mortality is generally low to moderate [7, 8, 11]. The intestinal damage caused by *C. suis* may predispose the piglet to other infections and co-infections with either viruses or bacteria are not uncommon [12–14]. Experimental infections have, for example, shown that co-infections with *C. suis* and *Clostridium perfringens* type A may worsen clinical signs and infected piglets may develop necrotic enteritis [13]. Other studies have shown that simultaneous infections with porcine rotavirus and *C. suis* may cause more extensive lesions in the intestine and a synergistic effect is suspected [14].

Both rotavirus and *C. suis* are globally considered as important gastrointestinal pathogens of suckling piglets [15]. A previous study has found both of these pathogens to be common in Swedish pigs aged one to 3 weeks [16]. In the 1980s, porcine rotavirus was demonstrated in 24% of the examined herds and in 14% of the examined litters in Sweden. In that study all the positive animals had steatorrhoea and rotavirus was not found in any of the piglets without clinical signs [16]. *C. suis* has previously been demonstrated in around 20% of piglets in Sweden [17].

In 1989 a new animal welfare law was implemented in Sweden which states that, for pigs, a maximum of 30% of the pen area may be slatted and, consequently, 70% of the floor of must be solid. Sows must also, by law, always be kept loose [18] and dry sows are often kept in deep litter straw systems. Overall, this type of housing may favour transmission of pathogens with a faeco-oral transmission route. Neither the rotavirus status nor the parasite status of pigs in Sweden has been scrutinised since the new animal welfare law was implemented in 1989. Therefore, the aim of this study was to document the prevalence of both rotavirus and *C. suis* in Swedish piglets, delivered by sows reared in deep litter systems and raised in pens with at least 70% solid floor, during the suckling and early weaning period.

## Materials and methods

### General information

By Swedish law, pigs are to be kept loose at all times including at farrowing. The slatted part of the floor must not exceed 30% of the total pen area, tails are non-docked and routine use of growth promotors has been banned since 1986 [18]. Dry sows are mostly kept in groups on deep litter straw and piglets are weaned at a minimum of 28 day of age. Age segregated rearing from birth to slaughter is commonly conducted whereby a group of sows enter a previously emptied and cleaned farrowing unit and the offspring are reared to market weight without mixing with other pigs. Pigs in Sweden are declared free from diseases on the former list A of the World Organisation of Animal Health (OIE), as well as from porcine respiratory and reproduction syndrome (PRRS), Aujeszky’s disease (AD), Salmonella and atrophic rhinitis (AR) [19–22].

### Experimental herds

In total, 81 sow herds were randomly selected from a register at the Swedish Board of Agriculture. The selected herds were sampled and a questionnaire regarding management routines was filled in. All selected herds had more than 80 sows and all but one of these herds conducted age segregated production from birth, thus emptying and cleaning each unit before entrance of new animals. The empty time between batches was 5.5 ± 4.5 days in the farrowing units and 6.2 ± 4.1 days in weaner units. Herd sizes and production results are shown in Table 1.

**Table 1:** Summary of herd sizes and production results of herds included in the study (mean ± SD)

			Farm classification		
Herds		ALL	Piglet producers	Satellites in multisite production	Farrow to finish
		(*n* = 81)	(*n* = 29)	(*n* = 9)	(*n* = 43)
Sows. mean	(n)	205 ± 211	185 ± 187	312 ± 116	243 ± 234
Sows. range	(n)	82–1200	100–1100	156–468	82–1200
Piglets, live born/year	(n)	25.7 ± 2.6	25.1 ± 4.8	26.0 ± 1.7	25.5 ± 3.1
Weaning age	(days)	36.5 ± 4.4	37.4 ± 4.3	34.9 ± 0.3	36.2 ± 4.7
Weaning weight	(kg)	10.2 ± 1.5	10.4 ± 1.7	9.8 ± 1.0	10.0 ± 1.4
Age at 25 kg	(days)	75.3 ± 5.1	74.9 ± 6.5	77.9 ± 5.3	75.1 ± 3.7
Piglet mortality					
Pre weaning	(%)	14.3 ± 4.1	15.3 ± 4.8	11.8 ± 3.1	14.0 ± 3.5
Post weaning	(%)	1.5 ± 1.0	1.8 ± 1.5	1.4 ± 1.0	1.4 ± 0.9

None of the herds used growth promotors and in 59 of the herds (73%), no routine treatments of pigs were carried out whatsoever. When sporadically piglets were diagnosed with diarrhoea, most often associated with *Escherichia coli* (*E. coli)*, they were individually treated following written instructions from the herd veterinarian. However, in 12 of the herds (15%; 10 integrated herds, 1 piglet producer and 1 satellite) all piglets were orally medicated with either antibiotics or 2500 ppm zinc oxide (ZnO) for one to 2 weeks following weaning. According to the antimicrobial policy of Sweden, laboratory tests confirming post weaning diarrhoea must have been made prior to initiating such treatments. In 14 of the herds (17%; 10 integrated herds and 4 piglet producers) all piglets were treated with 20 mg per kg body weight of the anti-protozoal agent toltrazuril (Baycox® vet, Bayer, Leverkusen, Germany) during the first week of life. Before initiating such treatment, the antimicrobial policy of Sweden states that *C. suis* must first be verified in the herd. Four of the integrated herds treated their piglets with both toltrazuril during the first week of life and antibiotics and/or ZnO post weaning. All herds in the study weaned the piglets at no earlier than 28 days and the mean time for weaning was 36.5 ± 4.4 days.

### Sample collection

In each of the 81 herds, ten litters were randomly selected. Ten faecal samples were collected from the pen floor of each selected litter. These ten samples were pooled into one sample per pen and kept cool until transported to the National Veterinary Institute in Sweden by mail. The samples were not cooled during the postal transport, which had a duration of less than 24 h. The same litters were sampled as the piglets were two, four and 6 weeks of age.

### Porcine rotavirus

Rotavirus was detected in the faecal samples using a sandwich enzyme-linked immunosorbent assay (ELISA), demonstrating virus antigen according to the instructions of the manufacturer (IDEA Rotavirus, Dako Ltd., Cambridgeshire, UK). The ELISA was based on polyclonal antibodies to rotavirus type A.

### Cystoisospora suis

*C. suis* was diagnosed using sedimentation according to Telemann. In short, one gram of faeces was suspended in 5 mL of a 5% acetic acid solution and shaken. The suspension was allowed to settle for one minute and then filtered through a sieve into a centrifuge tube. An equal amount of ether was then added, and the mixture was shaken before being centrifuged for one minute. The sediment was examined for coccidian oocysts and nematode eggs using light microscopy [23].

### Statistical analyses

All results are presented as mean ± standard deviation (SD) of the prevalence (%) of the herds or animals sampled. Paired student t tests and Spearman rank correlation tests between prevalence on herd levels were carried out (SAS Institute Inc., Cary, NC, USA). *P*-values < 0.05 were considered statistically significant.

## Results

### General information

All herds emptied and cleaned farrowing pens before introducing new sows. The 80 herds that effectuated age segregated rearing, emptied and cleaned whole units between batches. Out of these, 38 herds also disinfected the unit always or almost always, 22 herds disinfected regularly, and 20 herds disinfected never or almost never. All herds had extra heating in the farrowing pens (lamps, heated floor or both). Bedding was also used in all herds (straw in 79 herds and peat in two herds), occasionally complemented with saw dust. The quantity of bedding was medium to rich in all but two herds. The hygienic standard was assessed to be high in 74 herds and medium in seven herds.

### Experimental herds

At two weeks of age, 81 herds and a total of 791 litters were sampled. At four weeks of age, 74 herds and a total of 727 litters were sampled and, at six weeks, 72 herds and a total of 683 litters were sampled. In 72 out of the 81 farms, samples were retrieved on all three occasions. At every farm, the same litters were sampled on each occasion.

### Porcine rotavirus

**Fig. 1:**
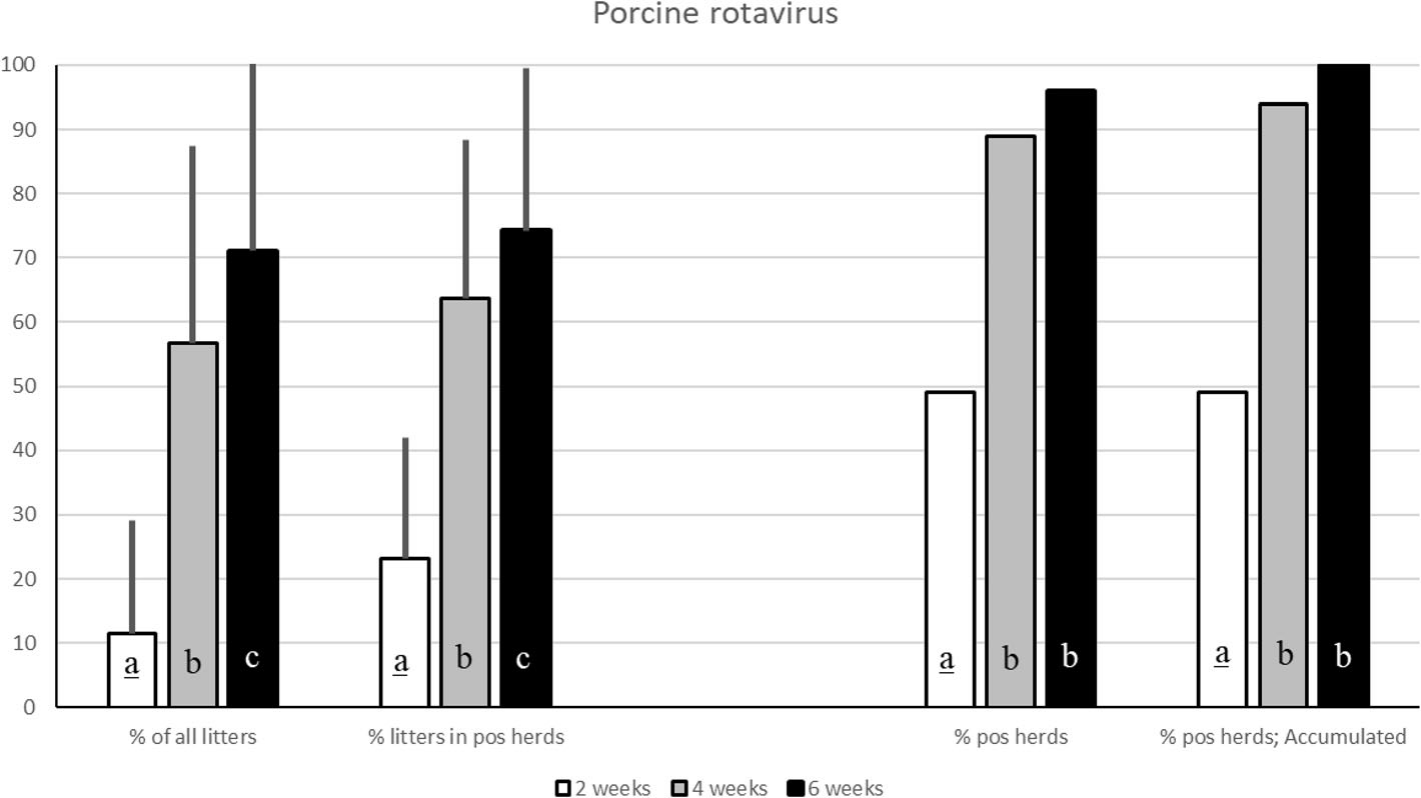
Prevalence of litters shedding rotavirus from two to six weeks of age.The overall prevalence of piglets shedding rotavirus from two to six weeks of age, as well as the prevalence of infected litters in herds where the virus was demonstrated. The true and the accumulated prevalence of rotavirus at herd level is shown to the right. Significant differences (*p*<0.05) between groups are indicated by different letters (if underlined *p*<0.001)

The overall prevalence of rotavirus in the examined herds was 11.4 ± 17.7% at 2 weeks, 56.8 ± 30.7% at 4 weeks and 71.1 ± 29.1% at 6 weeks of age. Rotavirus was found at all three sampling occasions in 30 out of the 72 herds (41.7%) and no herd was free from the virus at all three sampling occasions (Fig. 1).

At two weeks of age, 40 of 81 herds (49%) tested positive, with 23.1 ± 18.9% positive litters. At four weeks of age, 66 of 74 herds (89%) tested positive with 63.7 ± 24.6% positive litters. Finally, at six weeks of age, 69 of 72 herds (96%) tested positive for rotavirus with 74.2 ± 25.3% of the sampled pens being positive (Fig. 1).

### Cystoisospora suis

**Fig. 2:**
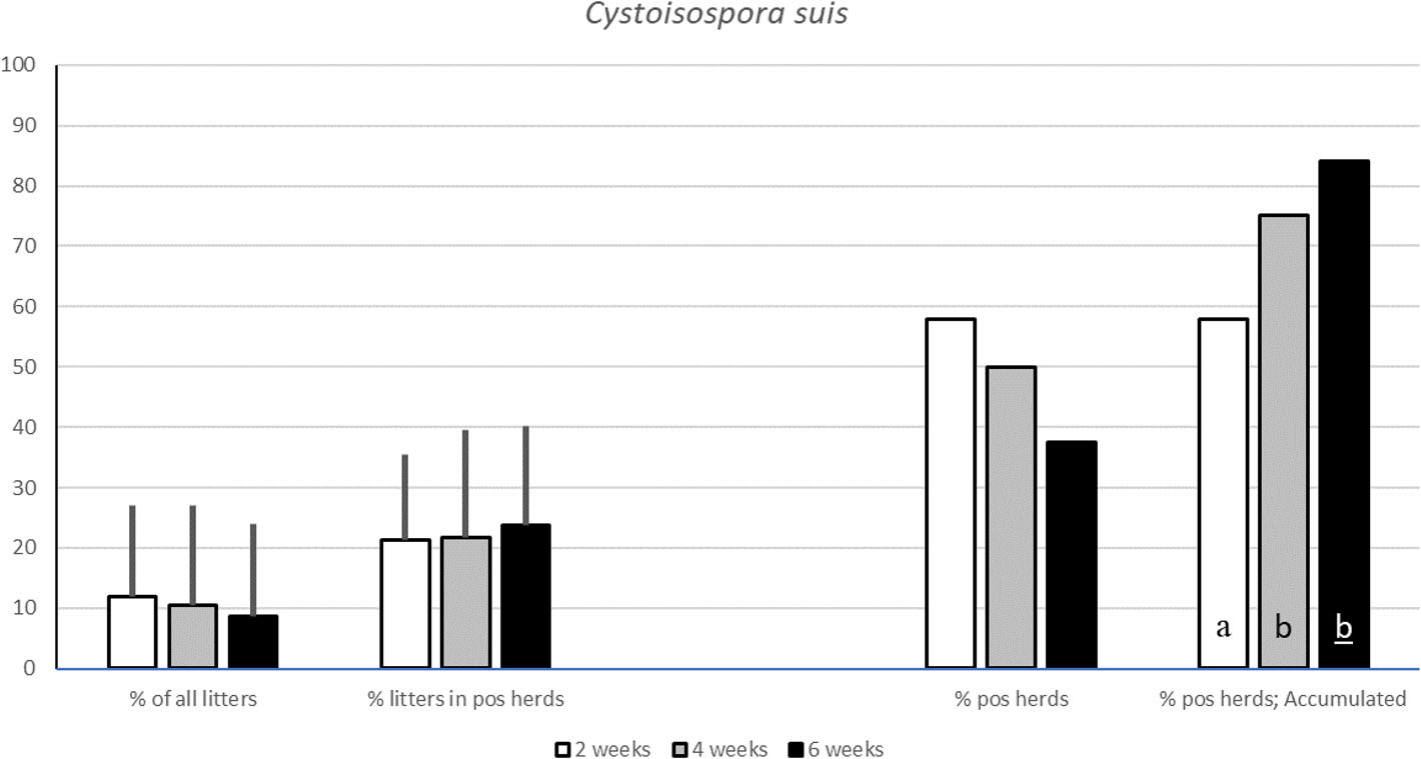
Prevalence of litters shedding C. suis from two to six weeks of age.The overall prevalence of piglets shedding C. suis from two to six weeks of age, as well as the prevalence of infected litters in herds where the parasite was demonstrated. The true and the accumulated prevalence of C. suis at herd level is shown to the right. Significant differences (*p*<0.05) between groups are indicated by different letters (if underlined p<0.01)

The overall prevalence of *C. suis* in the examined herds was 11.9 ± 15.1% at 2 weeks of age, 10.7 ± 16.7% at 4 weeks and 8.7 ± 15.3% at 6 weeks of age. *C. suis* was found at all three sampling occasions in eight of the 81 herds (11%), and in 11 of the 81 herds (15%) it was never detected (Fig. 2).

At two weeks of age, 47 out of 81 herds (58%) tested positive for *C. suis*. In these herds, *C. suis* was demonstrated in 21.4 ± 14.0% of the litters. At four weeks of age, 37 out of 74 herds (50%) tested positive with 21.8% ± 17.6% positive litters in these herds. Finally, at six weeks of age, 27 out of 72 herds (38%) tested positive with 23.8 ± 16.4% of the sampled litters being positive (Fig. 2).

### Other findings

*Balantidium coli* (*B. coli*) was found in 9.2% of the sampled litters at two weeks, 10.7% at four weeks and 25.8% at six weeks of age, respectively. Eggs from the parasitic nematodes *Ascaris suum (A. suum), Trichuris suis* (*T. suis*) and Strongylida species were also found in some of the samples from two weeks of age but these were considered transient intestinal passengers since patent infections of pigs at this young age are unlikely.

### Management factors

All but one herd employed age segregated rearing. One farrow-to-finish herd with 144 sows had a continuous production system. In that herd *C. suis* was demonstrated in 10, 10 and 40% of the litters at two, four and six weeks of age, respectively. Rotavirus was, in this herd, demonstrated in 0, 50 and 60% of the litters at two, four and six weeks of age, respectively. When dividing herds into piglet producers (*n* = 29), satellites in multisite production (*n* = 9), and farrow to finish herds (*n* = 43), no significant difference in prevalence of rotavirus nor *C. suis* was found (data not shown). As seen in Table 2, the herds that effectuated age segregated rearing had groups of sows farrowing at different time intervals, but the farrowing intervals did not appear to significantly influence the shedding of either rotavirus nor *C. suis*.

**Table 2:**
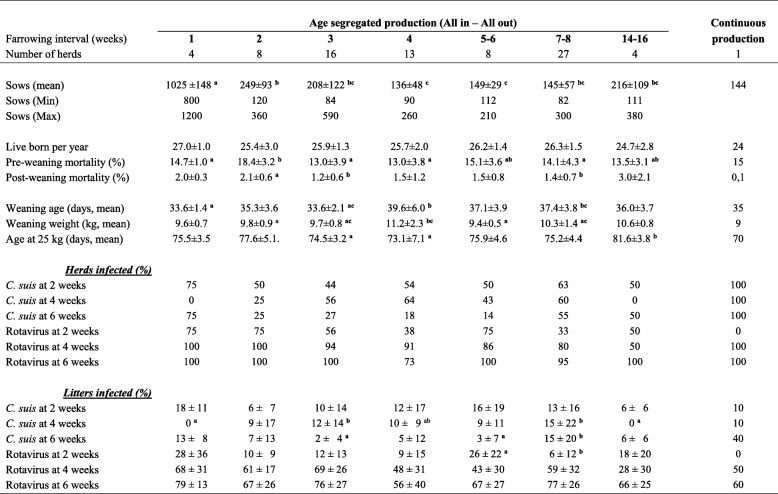
Prevalence of rotavirus and *C. suis* as well as productivity results in herds assorted with respect to farrowing intervals of the herds

Mean values within lines marked with different letters differ significantly (*p* < 0.05)

**Fig. 3:**
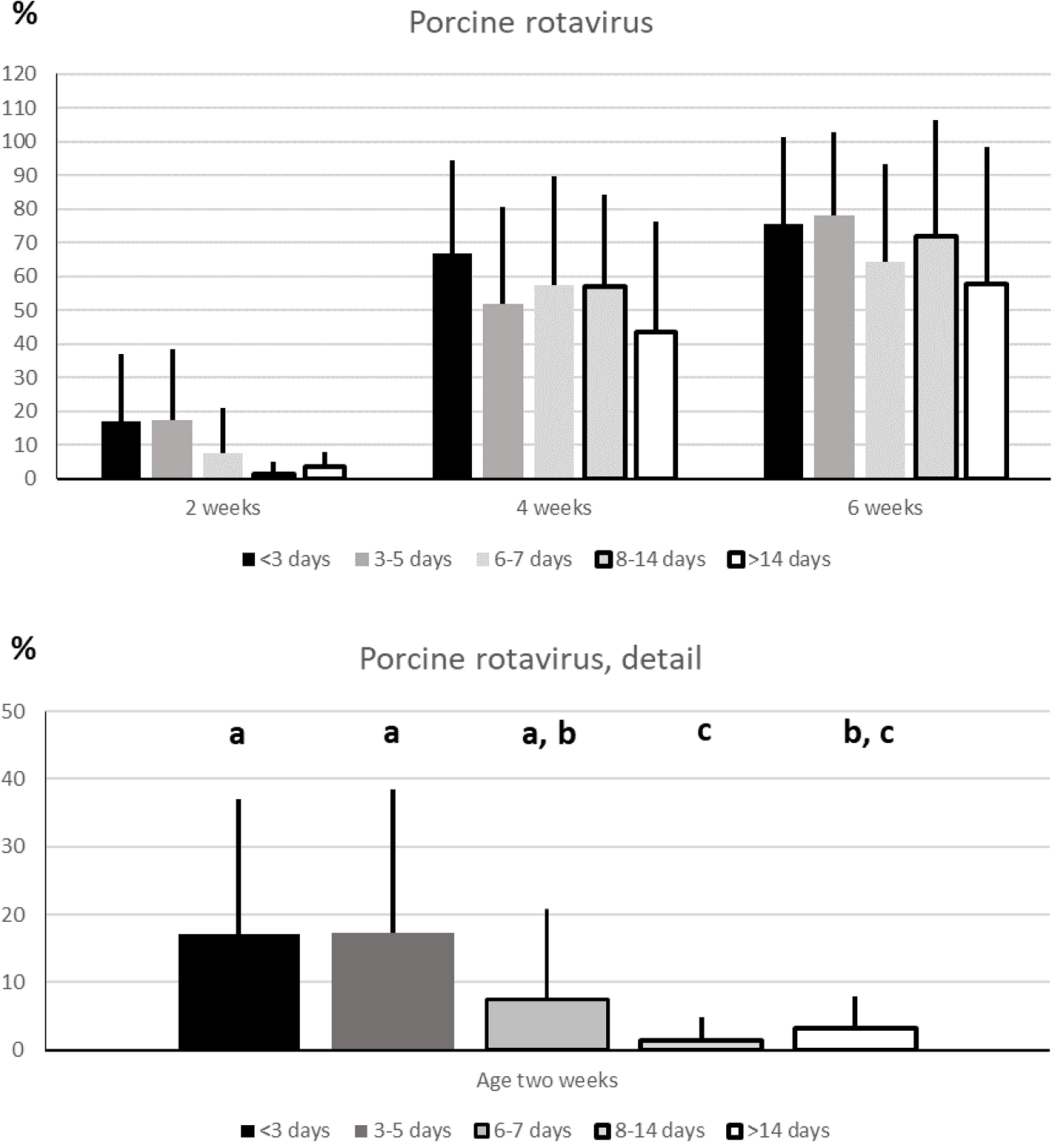
Rotavirus in comparison to the number of empty days between farrowing batches.The figures show the prevalence of rotavirus-positive litters aged two, four and six weeks, in comparison to the number of days when the farrowing pen was empty between farrowing batches. In the detail, categories with different letters differ significantly (*p*<0.05) from each other

**Fig. 4:**
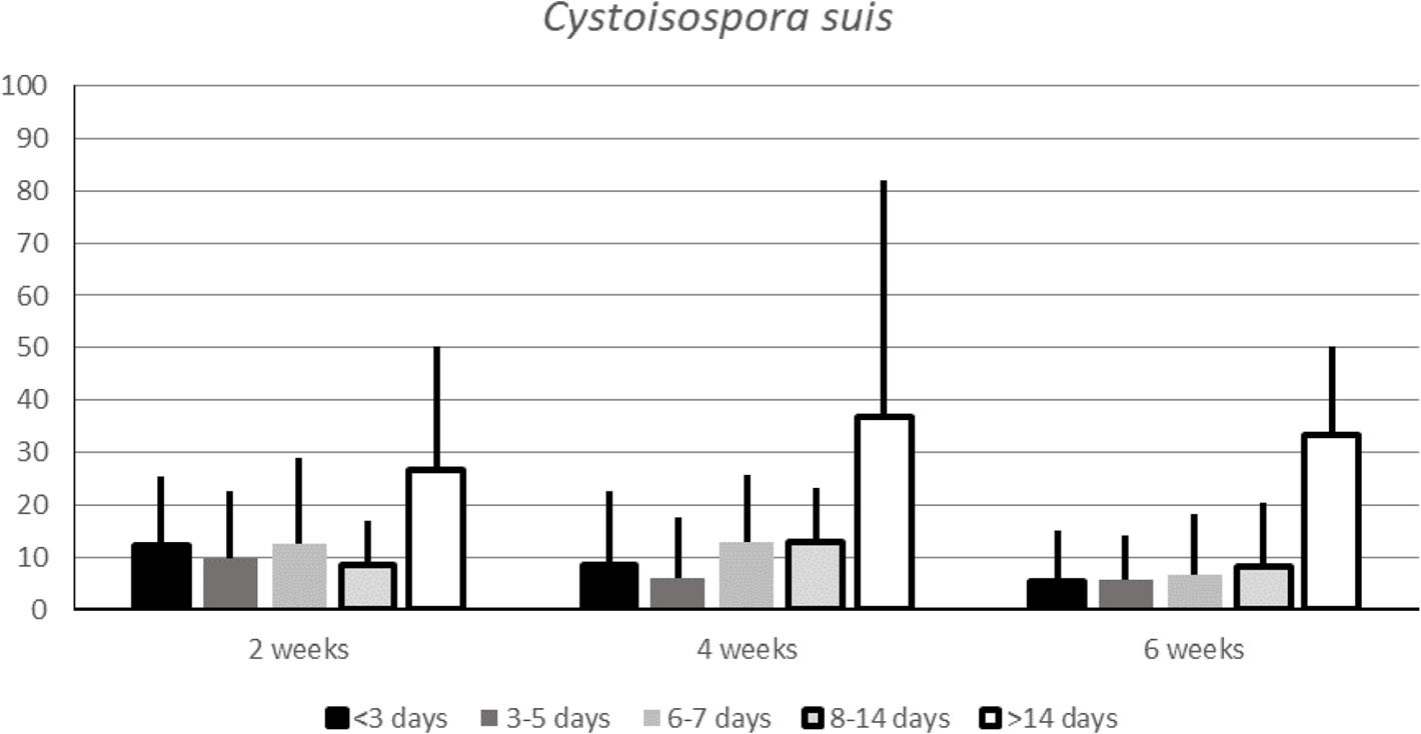
Cystoisospora suis in comparison to the number of empty days between farrowing batches.The figure shows the prevalence of C. suis-positive litters aged two, four and six weeks, in comparison to the number of days when the farrowing pen was empty between farrowing batches

All herds that employed age segregated rearing from birth, emptied and cleaned the farrowing units between batches. As seen in Fig. 3, the prevalence of litters infected with rotavirus at two weeks of age was lowest in seven herds with an empty period between farrowing batches of eight to 14 days (1.4 ± 3.5%). This number differed significantly from herds with less than three empty days between farrowing batches (*n* = 16; 17.1 ± 19.9%, *p* = 0.007) to herds with three to four empty days (*n* = 21; 17.4 ± 21.1%; *p* = 0.003) and to herds with five to seven empty days (*n* = 31; 7.4 ± 13.4%; *p* = 0.042). *P*-values for herds with five to seven empty days were 0.093 and 0.065 compared to herds with less than three and three to four empty days, respectively. By merging all herds with less than five empty days (*n* = 37; 17.3 ± 20.6% litters shedding rotavirus), they differed significantly to herds with five to seven empty days (*p* = 0.020) and to herds with eight to 14 empty days (*p* < 0.001). At the age of four and six weeks, no difference in prevalence of litters shedding rotavirus was observed. Regarding *C. suis*, no difference in prevalence of positive litters was correlated to the number of days between consecutive farrowing batches (Fig. 4).

In total, 14 of the herds administrated toltrazuril to the piglets during the first week of life. In one of these herds (7.1%), *C. suis* was never diagnosed. At the age of two weeks, *C. suis* was diagnosed in one litter, in one out of the 14 herds (7.1% infected herds; 0.7 ± 2.6 infected litters at herd level; 10% infected litters in the infected herd), which was significantly (*p* < 0.001) lower than in the non-treated herds. At the age of four weeks, *C. suis* was found in four herds (28.6% positive herds; 5.0 ± 9.1 infected litters at herd level; 17.5 ± 8.3% infected litters in infected herds), which was also significantly (*p* = 0.038) lower than in the non-treated herds. Finally, at six weeks of age *C. suis* was demonstrated in six of the 14 herds that treated their pigs with toltrazuril (42.9% positive herds; 7.1 ± 8.3% infected litter at herd level; 15.3 ± 4.7% infected litters in infected herds), which did not differ from non-treated herds. No significant difference between toltrazuril treated and non-treated piglets was seen regarding shedding of rotavirus, weaning age, weaning weight or age at 25 kg body weight. In total, 12 of the 81 herds administered either antimicrobials or ZnO in the feed for 14 days post weaning. At no occasion did the shedding of rotavirus or *C. suis* nor weaning age, weaning weight or age at 25 kg body weight differ significantly from non-treated piglets.

## Discussion

This study confirmed that porcine rotavirus must be regarded as an ubiquitous virus that can be expected to be present in almost every pig herd in Sweden. The study also demonstrated that the number of infected litters increased from birth to six weeks of age. Secondly, this study showed that *C. suis* frequently occurred in pig herds and that the number of infected litters was rather stable from two to six weeks of age. Thereby both these pathogens may play a role in intestinal disturbances during the suckling and post weaning periods.

Rotavirus has been a known cause of diarrhoea in young animals since the 1960s, with a worldwide distribution. It is considered an important cause of both impaired animal welfare and financial loss for pig farmers in Europe and porcine rotavirus was significantly more often demonstrated in neonatal piglets with diarrhoea than in control pigs in a recently published report [4, 24]. The increased shedding of rotavirus with age demonstrated in this study concurred a previous report which correlated this to a decrease in maternally derived antibodies [25]. Interestingly, there was significantly lower shedding of rotavirus in litters aged two weeks in herds that had a longer empty period between farrowing batches. This may indicate a decreased pathogen load during the neonatal period in herds with longer periods of emptiness between farrowing batches. However, this difference was not seen in the samples collected at four and six weeks of age. The chances of newborn piglets becoming infected increases as maternally derived antibodies wane. New piglets may become infected by virus present in the environment or from infected littermates and sows, and long empty periods between batches did not achieve long-term protection. This makes rotavirus as a possible contributor to post weaning diarrhoea as has been previously suggested [26]. In our study, an ELISA was used for detection of porcine rotavirus antigen. Studies have shown that this is not the most sensitive test to use when compared to molecular diagnostics such as polymerase chain reaction (PCR) for example, and it may be that the prevalence of rotavirus in our examined herds indeed was higher [27].

*C. suis* was first demonstrated in pigs in 1934, but it was not until the 1970s that the clinical importance of this pathogen was really recognised [11]. Since then it is considered an important parasite associated with diarrhoea in piglets all around the world and in all management systems [8, 9, 28]. The prevalence of coccidia varies greatly between countries, including between the Nordic nations [8, 17, 29]. In a large inter-Nordic study carried out in the 1980s, the prevalence of coccidia in piglets was around 20% in Sweden and Denmark, 31.8% in Iceland and less than 5% in Finland and Norway [17]. In this study, *C. suis* was common among piglets during the suckling and early weaning period. Despite that the number of herds where *C. suis* was demonstrated decreased from around 60% at two weeks of age to about 40% four weeks later, the prevalence of infected litters within a herd that remained infected, persisted over time. Still, the overall prevalence was around 50% lower when compared to earlier studies done in Sweden [16, 17]. This difference can probably be associated with the change to all in-all out production systems from birth to slaughter and the cleaning of facilities between consecutive batches of pigs, that was commonly induced as an effect of the ban of feed additives in Sweden in 1986 [18]. Piglets infected with *C. suis* shed oocysts in a cyclic fashion and shedding may vary greatly between individual animals in a litter [30, 31]. This makes diagnosis of *C. suis* a challenge and it has been recommended that at least two samplings ought to be carried out. A negative sample should also be followed with at least one new sampling one week later, as clinical signs and shedding of oocyst do not always occur simultaneously [8, 32]. A truly reduced prevalence of *C. suis* in our study, compared to the studies in the 1980s and 1990s, was supported by our study design where pooled samples collected at three separate occasions, two weeks apart, reduced the risk of false negative results at herd level. In our study, we used sedimentation and light microscopy to detect oocysts from *C. suis* in the faecal samples. Several diagnostic methods are available for the detection of *C. suis* and although the one used in this study was chosen for practical reasons it is not the most sensitive method. Highly sensitive and specific molecular diagnostic tools are available but are not practical or cost-effective to use in the field. A recent study comparing various methods for *C. suis* diagnosis found that autofluorescence microscopy of faecal smears is the most reliable, practical and economical diagnostic method to use in pig practice [32].

Something else that supports age segregated rearing could be a reduced risk of piglets becoming infected by ingesting sporulated oocysts in a contaminated farrowing pen. This despite high ambient temperatures in farrowing pens greatly favouring sporulation of *C. suis* oocysts which under the right conditions may become infective in 24 h [10]. Several studies have suggested that it is unlikely that sows are the main source of *C. suis* oocysts infecting piglets [9, 10, 16, 31]. Instead, a contaminated farrowing pen, resulting from poor cleaning between farrowing batches, or transmission between pens via fomites such as contaminated tools and boots of the staff, are the likely cause of oocysts infecting newborn piglets [31, 33, 34]. Thus, age segregated rearing with thorough cleaning and empty time between consecutive farrowing batches ought to minimise the risk of *C. suis* oocysts spreading between batches. Still, the prevalence of *C. suis* positive litters was not correlated with the number of days between consecutive farrowing batches which was surprising given that longer empty periods between batches ought to reduce the load of infective oocysts as has been previously described [33]. However, even if tools are unit-specific, boots are often used all over a herd and the staff are generally most active in the pig pens during the neonatal period, facilitating fomite transmission of *C. suis.* It should also be mentioned that cleaning and disinfection of the farrowing pens need to be done in such a way that it is effective against coccidia. Not all disinfectants used routinely by pig farms are effective against coccidia [35] and from our study it was noted that a large proportion of the participating farms used a disinfectant that was not effective against *C. suis.*

*B. coli,* another protozoan parasite, was found in 25,8% of the samples at six weeks of age. This parasite is generally considered non-pathogenic in pigs and infections are usually subclinical [2]. It is however a parasite with zoonotic potential and is considered an important parasite of pigs in some parts of the world [36]. As mentioned already in the results, eggs from *A. suum, T. suis* and Strongylida species found in piglets aged two weeks were defined as transients due to patent infections of these parasites being unlikely in piglets at this young age.

Intestinal disorders in piglets, mainly *E. coli* infections, were in more than 70% of the herds sporadic and treated at an individual level. The reduced incidence of *C. suis* following treatment with toltrazuril during the first week of life, as recorded in piglets aged two and four weeks in the 14 herds (17%)*,* proved the efficacy of the drug. Toltrazuril has been shown to be highly effective against cystoisosporosis by reducing the risk of diarrhoea, improving piglet health and reducing oocyst shedding. This has been seen under both experimental and field conditions [37–39]. However, the effect of this drug was no longer visible at six weeks of age, showing that toltrazuril solely will not solve clinical problems caused by *C. suis*. Instead this highlighted the importance of good hygiene and good management routines. This has also been shown in other studies [39]. In total, 12 herds (15%) that had experienced problems with *E. coli* associated diarrhoea at weaning, treated weaners with either antibiotics or ZnO in the feed. These treated herds performed equal to non-treated herds, indicating an effectiveness of the treatment. As expected, neither antibiotics nor ZnO had any influence at all on the shedding of *C. suis* or rotavirus, which emphasises the importance of having made a proper diagnosis before initiating treatment with such drugs [40].

## Conclusion

Porcine rotavirus was a common virus in piglets with an increased prevalence of infected litters from birth to six weeks of age. Likewise, *C. suis* was common in piglets with a remained prevalence of infected litters from birth to six weeks of age in the herds that tested positive for this parasite. Consequently, each of these pathogens may play a role in intestinal disturbances in piglets during the suckling and post weaning periods despite age segregated rearing, at least in systems with solid floors. Still, as this study was carried out in herds without reported problems with diarrhoea or poor weight gain, with high hygienic standard and with feed free from antibiotics, their role ought not to be overestimated. However, other factors such as concurrent infections, biosecurity and hygiene may influence the intestinal health in piglets. Hence, shortcomings in management may increase a negative impact of porcine rotavirus and *C. suis* with respect to health and productivity in pig herds.

## Abbreviations

*A. suum*: *Ascaris suum*
AD: Aujeszky’s disease
AR: Atrophic rhinitis
*B. coli*: *Balantidium coli*
*C. suis*: *Cystoisospora suis*
*E. coli*: *Escherichia coli*
ELISA: Enzyme-linked immunosorbent assay
OIE: World Organisation of Animal Health
PCR: Polymerase chain reaction
PRRS: Porcine respiratory and reproduction syndrome
*T. suis*: *Trichuris suis*
ZnO: Zinc oxide
